# ^18^F-Fluciclovine (^18^F-FACBC) PET imaging of recurrent brain tumors

**DOI:** 10.1007/s00259-019-04433-1

**Published:** 2019-08-15

**Authors:** Laure Michaud, B. J. Beattie, T. Akhurst, M. Dunphy, P. Zanzonico, R. Finn, A. Mauguen, H. Schöder, W. A. Weber, A. B. Lassman, R. Blasberg

**Affiliations:** 1 Molecular Imaging and Therapy Service, Department of Radiology, Memorial Sloan Kettering Cancer Center, 1275 York Avenue, Box 77, New York, NY 10065 USA; 2 Department of Medical Physics, Memorial Sloan Kettering Cancer Center, New York, NY USA; 3 Peter MacCallum Cancer Centre, Victoria, Australia; 4 Department of Epidemiology and Biostatistics, Memorial Sloan Kettering Cancer Center, New York, NY USA; 5 Department of Nuclear Medicine, Technical University, Munich, Germany; 6 Department of Neurology, Memorial Sloan Kettering Cancer Center, New York, NY USA; 7 Department of Neurology & Herbert Irving Comprehensive Cancer Center, Columbia University Irving Medical Center, New York, NY USA

**Keywords:** Glioma, PET, ^18^F-FACBC, ^18^F-Fluciclovine, ^11^C-methionine

## Abstract

**Purpose:**

The aim of our study was to investigate the efficacy of ^18^F-Fluciclovine brain PET imaging in recurrent gliomas, and to compare the utility of these images to that of contrast enhanced magnetic resonance imaging (MRI) and to [^11^C-methyl]-L-methionine (^11^C-Methionine) PET imaging. We also sought to gain insight into the factors affecting the uptake of ^18^F-FACBC in both tumors and normal brain, and specifically to evaluate how the uptake in these tissues varied over an extended period of time post injection.

**Methods:**

Twenty-seven patients with recurrent or progressive primary brain tumor (based on clinical and MRI/CT data) were studied using dynamic ^18^F-Fluciclovine brain imaging for up to 4 h. Of these, 16 patients also had ^11^C-Methionine brain scans. Visual findings, semi-quantitative analyses and pharmacokinetic modeling of a subset of the ^18^F-Fluciclovine images was conducted. The information derived from these analyses were compared to data from ^11^C-Methionine and to contrast-enhanced MRI.

**Results:**

^18^F-Fluciclovine was positive for all 27 patients, whereas contrast MRI was indeterminate for three patients. Tumor ^18^F-Fluciclovine SUVmax ranged from 1.5 to 10.5 (average: 4.5 ± 2.3), while ^11^C-Methionine’s tumor SUVmax ranged from 2.2 to 10.2 (average: 5.0 ± 2.2). Image contrast was higher with ^18^F-Fluciclovine compared to ^11^C-Methionine (*p* < 0.0001). This was due to ^18^F-Fluciclovine’s lower background in normal brain tissue (0.5 ± 0.2 compared to 1.3 ± 0.4 for ^11^C-Methionine). ^18^F-Fluciclovine uptake in both normal brain and tumors was well described by a simple one-compartment (three-parameter: V_b_,k_1_,k_2_) model. Normal brain was found to approach transient equilibrium with a half-time that varied greatly, ranging from 1.5 to 8.3 h (mean 2.7 ± 2.3 h), and achieving a consistent final distribution volume averaging 1.4 ± 0.2 ml/cc. Tumors equilibrated more rapidly (t_1/2_ranging from 4 to 148 min, average 57 ± 51 min), with an average distribution volume of 3.2 ± 1.1 ml/cc. A qualitative comparison showed that the rate of normal brain uptake of ^11^C-Methionine was much faster than that of ^18^F-Fluciclovine.

**Conclusion:**

Tumor uptake of ^18^F-Fluciclovine correlated well with the established brain tumor imaging agent ^11^C-Methionine but provided significantly higher image contrast. ^18^F-Fluciclovine may be particularly useful when the contrast MRI is non-diagnostic. Based on the data gathered, we were unable to determine whether Fluciclovine uptake was due solely to recurrent tumor or if inflammation or other processes also contributed.

**Electronic supplementary material:**

The online version of this article (10.1007/s00259-019-04433-1) contains supplementary material, which is available to authorized users.

## Introduction

Various amino acid PET tracers have been studied in brain tumors [1, 2]. They include [methyl-^11^C]-_L-_methionine (^11^C-Methionine), *O*-(2-[^18^F]-fluoroethyl)-_L_-tyrosine (^18^F-FET) and 6-[^18^F] -fluoro-L-DOPA (^18^F-DOPA). Amino acid accumulation in brain tumors is a function of both increased transport across tumor blood vessels and across tumor cell membranes due to overexpression of amino acid transporter systems [3]. Studies have shown that tumor detection by amino acid PET imaging is more sensitive than ^18^F-FDG PET imaging owing to the better tumor-to-normal brain image contrast seen with the amino acids. Also, with ^18^F-FDG, the appearance of tumor-associated inflammation can mimic that of tumor progression, but this is much less of a problem for amino acids [3–5]. Recent studies showed that amino acid PET imaging, including ^11^C-Methionine and ^18^F-FET, has greater accuracy than MRI for the evaluation of post-therapeutic effects [1] and of tumor recurrence. The European Association of Nuclear Medicine [3] and Neuro-oncology [2] recommend using amino acid PET rather than MRI for these indications. The diagnostic accuracies of current amino acid PET tracers (^11^C-Methionine and ^18^F-FET), however, remain suboptimal and the need for further improvement in neuro-imaging approaches persists [1–3, 6–8].

Pre-clinical studies have shown that ^18^F-Fluciclovine could be a potential PET tracer for brain tumor imaging [9], and the results from clinical studies evaluating ^18^F-Fluciclovine PET for primary staging of gliomas are encouraging [10–12]. Recently, Tsuyuguchi et al. showed in a preliminary study of six patients that ^18^F-Fluciclovine may provide better assessment than ^11^C-Methionine for the initial detection of glioma [13]. Furthermore, ^18^F-Fluciclovine may be a promising tracer to detect high grade recurrent gliomas [14].

The objective of the present study was to investigate the efficacy of ^18^F-Fluciclovine brain PET imaging in the setting of recurrent or progressive disease and to compare it to contrast-enhanced MRI imaging and to ^11^C-Methionine brain PET imaging.

## Methods

### Patients

This prospective clinical study was performed after Institutional Review Board approval (Memorial Sloan-Kettering Cancer Center IRB#03–028). Twenty-seven patients who underwent ^18^F-Fluciclovine brain PET for suspicion of recurrent or progressive gliomas between August 2004 and January 2008 were recruited under two different protocols (16 in the first, 11 in the second) with different funding sources (NCI and an internal source). The inclusion criteria, however, were identical and the data acquisition procedures were very similar with the primary difference being that the ^11^C-methionine study was left off of the second protocol. All patients fasted for at least 4 h before PET imaging. Written informed consent was obtained from all patients.

### Synthesis of ^18^F-Fluciclovine and ^11^C-methionine

Clinical-grade ^18^F-labeled Fluciclovine and ^11^C-labeled methionine were produced by the Radiochemistry and Molecular Imaging Probes Core at MSKCC.

### ^18^F-Fluciclovine and ^11^C-methionine PET image acquisition

Following a 1-min IV infusion of 370 MBq of ^18^F-Fluciclovine, dynamic images of the brain were acquired in 2D mode (i.e. with septa) for 45 min on a GE Advance PET (first 21 patients) or a GE Discovery STE PET/CT (last 6 patients) scanner. The patients were subsequently removed from the scanner but returned up to three times for 20-min static images of the brain between 90 and 240 min post injection. No adverse effects were reported following the injection of ^18^F- Fluciclovine.

For a subset of patients who agreed to the procedure, a heated-hand arterialization of the venous blood was performed using chemical heating pads. Starting at 30 s post initiation of the ^18^F-Fluciclovine infusion, ~1-mL blood samples were acquired approximately every 20 s, with the precise time (synchronized to the PET computer’s clock) recorded for each sample. As time progressed, the spacing between samples was increased (at most, a total of 25 samples were acquired for each patient). The later samples were taken at the same times as the respective late static imaging sessions (one sample at or soon after each session). For each blood sample, a portion was used to obtain plasma, and aliquots of whole blood and plasma were assayed for ^18^F activity concentration using a calibrated gamma counter [LKB Wallac]. This procedure in many cases failed (e.g., hand insufficiently heated) but was judged to have been successful in 8 (out of 20 attempted) patients, whose resulting blood time-activity curves achieved a peak SUV greater than 8.

For 16 of the patients, ^11^C-Methionine PET was performed on the same day, 2.5 or more hours prior to the initiation of the ^18^F-Fluciclovine PET imaging. In these scans, dynamic images of the brain were acquired over 45 min, following a 1 min IV infusion of 370 MBq of [^11^C-methyl]-L-methionine. No adverse effects were reported after the injection of ^11^C-Methionine.

All PET images were reconstructed with an iterative ordered subsets expectation maximization (OSEM) algorithm available from the PET camera manufacturer. All emission scans were accompanied by a 2- to 5-min germanium-68 transmission scan (on the GE Advance) or low dose CT scan (on the GE DSTE) for attenuation correction purposes. Manufacturer-provided corrections for scatter, randoms and detector inhomogeneity were also applied.

### Image analysis

All ^18^F-Fluciclovine and ^11^C-Methionine visual and SUV analyses were conducted using the Hermes HybridViewer imaging software (Hermes Medical Solutions, Stockholm, Sweden) based on a summed 25–45 min PET static image set registered to a contrast-enhanced MR (or in one case CT) image acquired within 30 days of the PET image. We chose to base our analysis upon a summed 25–45 min image because the uptake was relatively stable over this time period; it was the latest time consistently acquired across patients for both ^18^F- Fluciclovine and ^11^C-Methionine and it was similar to time points used in other studies of ^18^F-Fluciclovine uptake into brain tumors [10, 12].

The reader, an experienced nuclear medicine physician (LM), interpreted the scans visually and classified them as positive (high or low tumor uptake) or negative (no uptake), and manually defined the borders of the tumor on each of the scans (independently and blinded to the patient clinical and imaging outcomes). Tumor SUVmax of each patient was recorded along with its ratio to the SUVmeans taken from two different normal-brain reference regions. One reference region was placed over the contralateral normal brain [3], and the other placed within the cerebellum. The degree of overlap between all paired modality combinations (MRI-Fluciclovine, MRI-Methionine and Fluciclovine-Methionine) were assessed using Sorensen-Dice coefficients [15, 16]*.*

For the kinetic analysis, time-activity curves describing the ^18^F-Fluciclovine uptake were derived from large regions of interest placed within the normal cerebellum and small (~1–3 cc) regions covering the most intense uniform portion of the lesion, both initially based solely on their appearance within the summed 25–45 min image. The uptake within the tumor region was also assessed visually at all other time-points to assure homogeneity within the region and throughout the entire time-course of the study. Adjustments to the volume were made if this criterion was not met. We chose not to do modeling on the contralateral normal brain out of concern that the regions would contain a mixture of white and gray matter thereby violating the homogeneous tissue assumption implicit in the use of a compartmental model.

### Kinetic analysis of dynamic ^18^F-Fluciclovine PET images

The static ^18^F-Fluciclovine PET images were each registered to the summed 25–45 min frame of the dynamic image set using a rigid-body transform that maximized a normalized cross-correlation target function. The resultant resampled image sets were then appended to the end of the initial 45-min dynamic series resulting in a single dynamic PET image set extending up to 4-h in duration. All PET and blood activity concentration measures were converted to standard uptake values (SUVs) normalized by patient body mass.

Blood sample data was fitted to a sum of three exponentials convolved with a square pulse mimicking the ^18^F-Fluciclovine infusion. The resulting sum of exponentials was then used as the input function, *I*, when fitting the tumor and normal cerebellum time-activity tissue curves, *T*, using a nonlinear search algorithm that minimized the weighted least-squares difference between the data and the following model equation:

$$ T={V}_b\;I+{k}_1{e}^{-{k}_2t}\ast I $$

where * denotes convolution, *t* is time, V_b_ is the blood volume and k_1_ and k_2_ are the first-order rate constants describing the transport of ^18^F-Fluciclovine between the plasma and the tissue.

During the fitting process the above equation was averaged over the durations of the individual PET frames from which *T* was determined. The weights used in the fitting were chosen based on an estimate of the total counts contributing to the tissue measurement (i.e. a function of the frame duration and the tissue activity concentration). Models involving an additional exponential were also evaluated based on a comparison of their Bayesian Information Criterion (BIC) values [17].

### Reference standard

To establish the ground truth, tumor pathology results were used whenever a biopsy or a surgery was performed. In patients for which no tissue samples were available, the clinical and imaging follow-up was used.

### Statistical analysis

All statistical comparisons of the 25–45 min uptake metrics, and ratios thereof, were conducted using a Spearman correlation. All tumor volume comparisons made use of a Wilcoxon paired test. Kaplan-Meier survival analysis was performed and the relation between risk of death and tumor SUVmax (Tmax), tumor SUVmax to contralateral SUVmean (Tmax/Co_mean), tumor SUVmax to cerebellar SUVmean (Tmax/Ce_mean) and tumor volume were evaluated using a Wald test in a Cox model. All analyses were performed using R version 3.5.0 (www.r-project.org). A *p* value of less than 0.05 was considered statistically significant for all analyses.

## Results

### Patients

Twenty-seven ^18^F-Fluciclovine PET studies were performed. Patient characteristics are summarized in Table 1. All patients had contrast-enhanced conventional imaging (26 MRI and 1 CT) within 30 days of FACBC PET (mean 9 days) to evaluate recurrent or progressive disease: based on the MRI results 22 patients were considered to have recurrence or progressive disease, two patients stable disease and three patients had equivocal results by MRI.

**Table 1:** Patients characteristics

Patient	Sex	Age years	Initial tumor type	Initial grade	Previous surgery	Previous RT	Previous ChemoT	Previous other ttt	Delay months	MRI (or CT) results, within 30 days of FACBC PET	Recurrence histology
1	M	31	Astrocytoma	II	1	1	0	0	92.05	+ recurrent/residual tumor	High grade glioma
2	M	62	Astrocytoma	II	1	1	1	0	20.28	+ progression	GBM
3	F	50	GBM	IV	1	1	1	1	9.90	+ residual tumor	–
4	M	53	Oligodendroglioma	III	1	1	0	0	10.49	+ recurrent/residual tumor (CT)	–
5	F	47	GBM	IV	1	1	0	1	2.76	Equivocal: (1)	GBM
6	M	54	Astrocytoma	II	1	0	1	0	136.96	+ progression	–
7	M	46	Astrocytoma	III	1	1	1	0	22.91	+ progression	High grade astrocytoma
8	M	71	GBM	IV	1	1	1	0	15.25	Stable disease	–
9	M	49	GBM	IV	1	1	1	0	30.25	Equivocal: (1)	–
10	M	40	Astrocytoma	III	1	1	1	1	20.35	+ viable tumor	–
11	M	61	GBM	IV	1	1	1	0	7.89	+ progression	–
12	M	27	Astrocytoma	III	1	1	1	1	10.19	+ progression	–
13	M	55	GBM	IV	1	1	1	1	13.55	+ progression	GBM
14	F	41	Oligoastrocytoma	II	1	1	1	0	24.92	+ progression	–
15	M	59	Astrocytoma	III	1	1	1	0	48.2	+ recurrence	GBM
16	F	60	GBM	IV	1	1	1	0	12.46	+ progression	–
17	M	55	Astrocytoma	II	1	1	0	0	6.18	+ progression	GBM
18	F	61	Oligodendroglioma	II	1	1	1	0	85.38	+ tumor bilateral	–
19	M	37	GBM	IV	1	1	1	0	11.84	+ progression	–
20	M	47	Astrocytoma	II	1	1	1	0	68.15	+ progression	Low grade glioma
21	F	44	GBM	IV	1	1	1	1	19.79	+ progression	GBM
22	M	45	Astrocytoma	III	1	1	1	0	15.58	+ progression	GBM
23	M	54	GBM	IV	1	1	1	1	6.31	Stable disease	–
24	F	57	Oligodendroglioma	II	1	1	1	1	292.8	+ progression	–
25	F	67	GBM	IV	1	1	1	0	67.20	Equivocal: (2)	GBM
26	F	53	Oligodendroglioma	II	1	1	1	0	155.77	+ progression	Low grade glioma
27	M	57	GBM	IV	1	1	1	1	15.35	+ recurrence	GBM

Patients 1–16 were scanned with ^18^F- Fluciclovine and 11C-Methionine PET imaging. Delay months = Time between initial surgery and PET imaging. Other ttt = immunotherapy / poly ICLC /Interleukin-4-Pseudomonas exotoxin chimeric fusion protein. 1 = performed, 0 = not performed, + = positive, (1) = recurrence or radionecrosis, (2) = stable or low growing

All patients were considered to have an active tumor based upon the clinical and/or MRI assessment at the time of ^18^F-Fluciclovine imaging. Thirteen patients had a biopsy or a surgical resection after the ^18^F-Fluciclovine imaging providing a proof of the recurrence. Pathology at this stage revealed: nine patients with GBM (5 initially with GBM, 2 initially with astrocytoma grade III and 2 initially with astrocytoma grade II). In addition, there were two low grade gliomas (initially oligodendroglioma grade II and astrocytoma grade II), one high grade astrocytoma (initially an astrocytoma grade III), one high grade glioma (initially an astrocytoma grade II) (Table 1). In 13 other patients without a second pathological diagnosis (5 patients had initial low-grade gliomas and 8 had high grade gliomas), the diagnosis of recurrent/progressive disease was based on clinical follow up. For one patient (patient 4, with an initial oligodendroglioma grade III), there was no medical record after the ^18^F-Fluciclovine PET, but MRI and PET imaging suggested tumor recurrence. That patient died 9 months later, suggesting an active tumor.

**Fig. 1:**
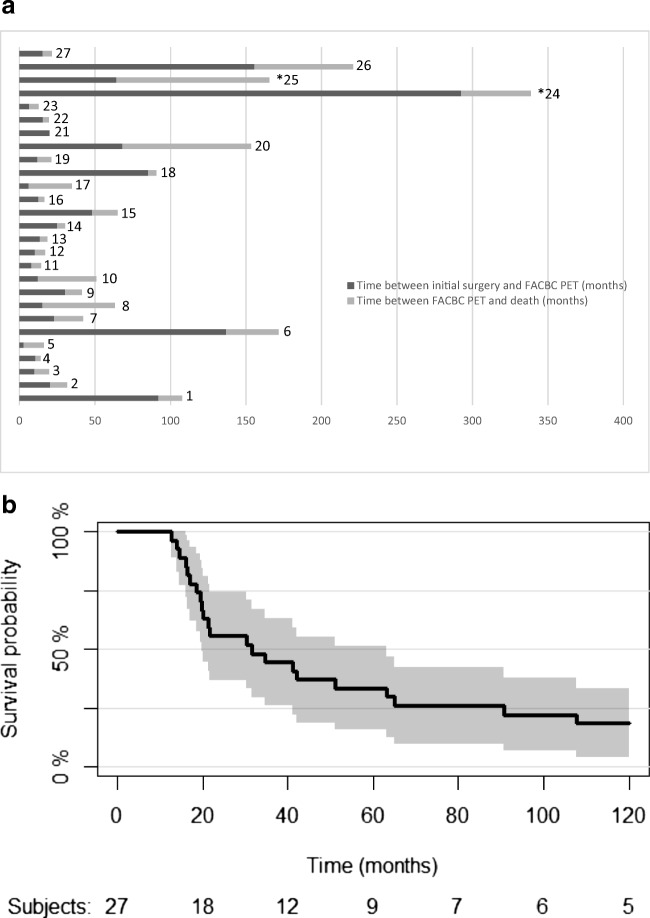
Patient survival. **a.** Patients’ duration of disease (*Patients 24 and 25 last seen date instead of death) expressed as the time between initial surgery and Fluciclovine PET and as time between Fluciclovine PET and death. **b.** Kaplan Meier survival. The median overall survival is 31.6 months. The 5-year overall survival rate is 33% (95% CI: 16–51%)

Twenty-five patients are known to have died during the follow up period. For two patients (patients 24 and 25), only a single post-PET imaging follow-up note was available; both had clinical disease progression at that time (Fig. 1). The median overall survival was 31.6 months and the 5-year overall survival rate was 33% (95% CI: 16–51%).

### ^18^F-Fluciclovine and ^11^C-methionine uptake: Analysis of 25–45 min static images

**Fig. 2:**
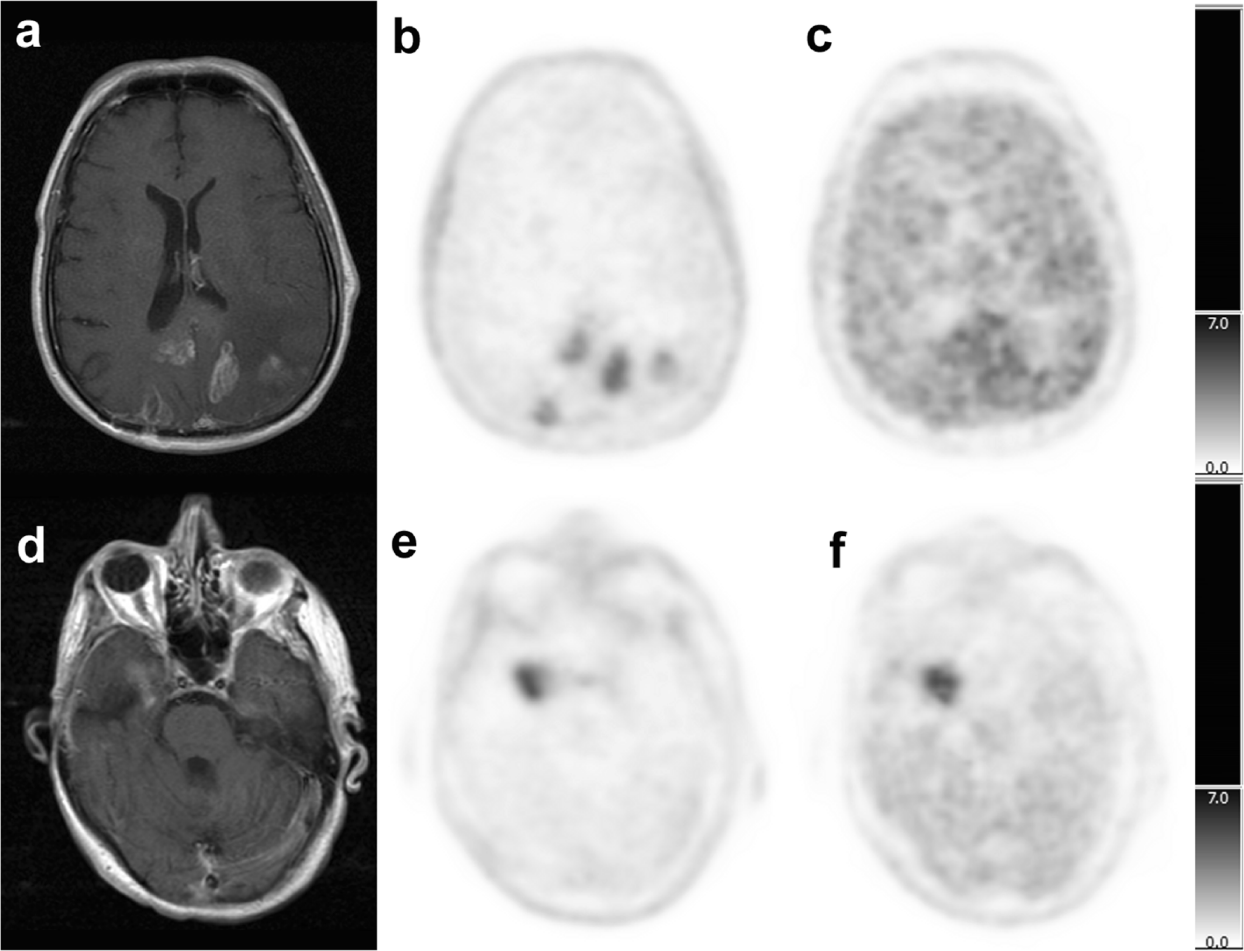
Patient examples. Top row - Patient 11 imaging. Male 61 years old. Primary tumor: GBM grade IV treated by surgery followed by adjuvant RT and chemotherapy (Temozolomide). Four months after the surgery, clinical suspicion of progression. MRI (*image a. Axial*) evocative of multifocal tumor progression. ^18^F-Fluciclovine PET (*image b. Axial*): multifocal tumor uptake SUVmax 4.5, ratio tumor SUVmax/ Contralateral SUVmean 6.9 and ratio tumor SUVmax/ Cerebellum SUVmean 8.1. ^11^C-Methionine PET (*image c. Axial*): visual very low tumor uptake SUVmax 5.09, ratio tumor SUVmax/ Contralateral SUVmean 2.5 and ratio tumor SUVmax/ CerebellumSUV mean 2.5. Progression treated by protocol RAD-Iressa. The patient died 7 months after. Autopsy: GBM complicated by bronchopneumonia. Bottom row - Patient 15 imaging. Male 59 years old. Primary tumor: right temporal anaplastic astrocytoma grade III treated by surgery followed by RT and chemotherapy (Temozolomide). Four years after: headaches increased. MRI (*image d. Axial*) evocative of right temporal tumor recurrence. ^18^F-FACBC PET (*image e. Axial*): visual high tumor uptake SUVmax 3.13, ratio tumor SUVmax/ Contralateral SUVmean 13 and ratio tumor SUVmax/ Cerebellum SUV mean 8.5. ^11^C-Methionine PET (*image f. Axial*): visual high tumor uptake SUVmax 2.96, ratio tumor SUVmax/ Contralateral SUVmean 5.6 and ratio tumor SUVmax/ Cerebellum SUV mean 3.3. Progression treated by Avastin, surgery and chemotherapy (Temozolomide). The histology report was evocative of a GBM. The patient died 1 year later

The tumor uptake and tumor-to-normal brain ratio results are summarized in Table 2, and representative PET scans are shown for two patients (Fig. 2). All 27 tumors were clearly delineated with ^18^F-Fluciclovine PET. ^18^F-Fluciclovine tumor uptake ranged from 1.5 to 10.4 SUVmax (average ± standard deviation 4.5 ± 2.3), Co_mean ranged from 0.14 to 1.08 (0.4 ± 0.2), Ce_mean ranged from 0.2 to 1.75 (0.6 ± 0.3) and Tmax/Co_mean and Tmax/Ce_mean ratios from 3.6 to 23.8 (10.8 ± 4.5) and from 4.3 to 15.2 (7.9 ± 2.8), respectively***.*** In three patients with equivocal MRI results (patients 5, 9 and 25), ^18^F-Fluciclovine PET confirmed tumor progression (all GBM, including two biopsy-proven after imaging). Two patients had visually low ^18^F-Fluciclovine tumor uptake. One of these two patients (patient 20) had an astrocytoma grade II at time of diagnosis, and five years later, clinical suspicion of recurrence. The MRI showed recurrent disease with a small tumor volume (4.4 cm^3^).^18^F-Fluciclovine tumor SUVmax and tumor/brain ratio were low at 2.06 and at 3.6 and 4.3 for ratios to contralateral and cerebellum, respectively, and PET tumor volume was 2.5 cm^3^(Tables 1 and 2). A resection of this tumor after Fluciclovine imaging showed a recurrent low-grade glioma. Eleven years after initial presentation (6 years after the ^18^F-Fluciclovine PET scan), this patient progressed again. Re-resection of the recurrent tumor showed a high-grade astrocytoma. The second patient (patient 23) had an initial GBM. The MRI showed stable disease and a small tumor volume (5.7 cm^3^). ^18^F-Fluciclovine tumor SUVmax and tumor-to-cerebellum ratio were low (2.4 and 4.5, respectively), but the ratio of tumor to contralateral normal brain was relatively high (7.1). The tumor was not re-resected, and the patient died 6 months after the PET imaging.

**Table 2:** PET imaging results (summed 25–45 min PET static image)

Patient	MRI (or CT)		^18^F-FACBC PET						^11^C-Methionine						Reference standard
	Results	V cm3	Visual	Tmax	Co mean	Tmax/Co mean	Tmax/Ce mean	V cm3	Visual	Tmax	Co mean	Tmax/Co mean	Tmax/Ce mean	V cm3	
1	+	12.8	U	2.69	0.47	5.7	4.6	18.8	U	4.18	1.23	3.4	3.1	20.5	H +
2	+	73.5	U	5.02	0.66	7.6	8.0	35.4	U	6.62	1.95	3.4	3.1	32.9	H +
3	+	80	U	4.16	0.35	11.9	7.7	74.3	U	4.90	1.42	3.5	2.8	79.4	FU
4	+ (CT)	25.1	U	7.13	0.51	14.0	15.2	37.2	U	5.23	1.4	3.7	2.5	38.2	?
5	?	20.0	U	5.90	0.53	11.1	10.2	25.0	U	6.90	1.37	5.0	4.6	38.1	H +
6	+	57.9	U	2.54	0.27	9.4	5.1	6.6	LU	3.53	1.02	3.5	3.1	2.8	FU
7	+	6.3	U	4.67	0.28	16.7	10.4	5.2	U	5.39	1.14	4.7	4.0	3.3	H – then +
8	Stable	1.1	U	2.23	0.38	5.9	4.8	1.7	LU	2.95	1.17	2.5	1.5	0.3	FU
9	?	1.1	U	2.56	0.3	8.5	6.1	1.1	LU	2.76	1.16	2.4	1.9	0.6	FU
10	+	72.6	U	5.39	0.47	11.5	5.6	57.7	U	6.52	1.77	3.7	2.8	45.5	FU
11	+	59.1	U	4.51	0.65	6.9	8.1	65.2	LU	5.09	2.03	2.5	2.5	33.4	FU
12	+	16.5	U	8.83	1.08	8.2	5.0	27.8	U	8.00	1.99	4.0	2.7	13.6	FU
13	+	22.9	U	8.39	0.45	18.6	12.3	39.9	U	10.18	1.19	8.6	6.0	28.8	H +
14	+	30.8	U	2.46	0.42	5.9	5.0	14.3	LU	3.22	1.16	2.8	1.9	7.3	FU
15	+	6.1	U	3.13	0.24	13.0	8.5	3.8	U	2.96	0.53	5.6	3.3	5.3	H +
16	+	32.0	U	1.99	0.15	13.3	9.5	63.3	LU	2.22	0.48	4.6	3.3	49.0	FU
17	+	19	U	3.74	0.24	15.6	12.5	21.1							H +
18	+	31.4	U	3.33	0.14	23.8	10.4	19.6							FU
19	+	14.3	U	1.48	0.18	8.2	7.4	9.9							FU
20	+	4.4	LU	2.06	0.57	3.6	4.3	2.5							H +
21	+	46.7	U	5.18	0.53	9.8	5.6	25.6							H +
22	+	11.8	U	10.44	0.74	14.1	7.7	11.5							H +
23	Stable	5.7	LU	2.41	0.34	7.1	4.5	6.7							FU
24	+	34.9	U	4.81	0.4	12.0	9.6	41.1							FU
25	?	1.30	U	5.55	0.69	8.0	5.3	1.4							H +
26	+	9.84	U	3.52	0.38	9.3	7.5	9.3							H +
27	+	139.7	U	6.60	0.54	12.2	9.4	153.7							H +

P = patients, V = volume, Tmax = tumor SUVmax, Co mean = contralateral SUVmean, Ce mean = cerebellum SUVmean, + = positive, ? = equivocal, U = uptake, LU = low uptake, H = histology, FU = follow up

We did not attempt to determine the correlation between tumor uptake and tumor grade because too few patients had a biopsy or a surgical resection at the time of the ^18^F-Fluciclovine PET imaging (and therefore there was no histology on which to base an assessment of grade) and because too few patients had low-grade tumors (2/13 among the patients with re-resection and 5/14 based on initial grading).

**Fig. 3:**
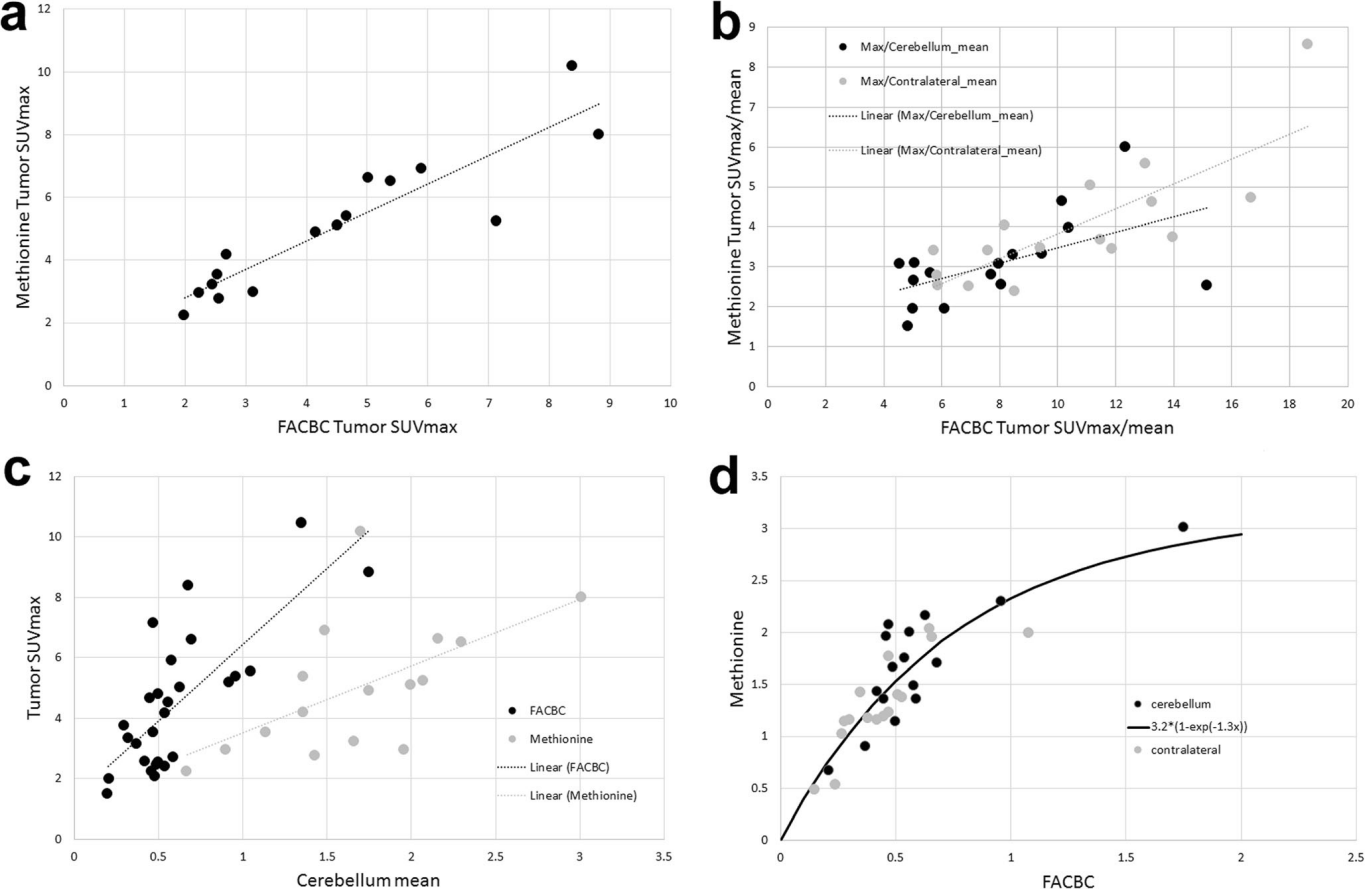
Comparison of various uptake metrics for ^18^F-Fluciclovine and ^11^C-Methionine. All measured values are an average over the 25 to 45 min time frame. **a.** Correlation between maximum tumor SUV’s for the two PET racers. **b.** Essentially the same comparison as in A but this time the SUVmax values have been normalized by either the normal cerebellum (*black circles*) or normal contralateral (*gray circles*) means. **c.** Shows the correlation between the tumor SUVmax and the same patient’s cerebellar mean for ^18^F-Fluciclovine (*black circles*) and ^11^C-Methionine (*gray circles*). **d.** Relationship between the brain uptake for ^18^F-Fluciclovine and the same patient’s brain uptake of ^11^C-Methionine in the normal cerebellum (*black circles*) and in the normal contralateral brain (*gray circles*)

Sixteen patients had both ^18^F-Fluciclovine and ^11^C-Methionine PET imaging. Six of 16 patients had low tumor uptake of ^11^C-Methionine, and extremely low tumor contrast (Fig. 2A). ^11^C-Methionine PET tumor SUVmax ranged from 2.2 to 10.2 (average 5.0 ± 2.2), Co_mean ranged from 0.48 to 2.03 (1.3 ± 0.4), and Ce_mean ranged from 0.67 to 3.1 (1.7 ± 0.6). SUVmax values were lower with ^18^F-Fluciclovine than with ^11^C-Methionine (*p* = 0.03), but there was a very high correlation between tumor ^18^F-Fluciclovine and ^11^C-Methionine SUVmax values (r = 0.92) (Fig. 3a). This correlation decreased somewhat when the tumor values were normalized by either cerebellar (r = 0.52) or contralateral (r = 0.76) normal brain mean values (Fig. 3b).

Visual analysis clearly showed higher tumor-to-normal brain image contrast with ^18^F-Fluciclovine than with ^11^C-Methionine, due to the lower background (lower normal brain uptake) in the ^18^F-Fluciclovine studies (Fig. 2a and 2b). Uptake in the contralateral brain was about 30% below that of the cerebellum, for both ^18^F-Fluciclovine and ^11^C-Methionine, but the two regions were highly correlated across patients (^18^F-Fluciclovine r = 0.78 and ^11^C-Methionine r = 0.88). The higher contrast of the ^18^F-Fluciclovine images was quantitatively confirmed when we compared the Tmax/Co_mean and Tmax/Ce_mean ratios between ^18^F-Fluciclovine and ^11^C-Methionine. Both ratio values were significantly higher with ^18^F-Fluciclovine (*p* < 0.0001) (Fig. 3c).

An intra-patient comparison of ^18^F-Fluciclovine and ^11^C-Methionine uptake in normal brain (contralateral and cerebellar combined) showed the uptake of the two tracers to be functionally, though perhaps not completely linearly related (Supplement S-4), with ^11^C-Methionine uptake over three times that of ^18^F-Fluciclovine. A slight but persistent negative correlation of normal brain uptake and patient age was seen for both ^18^F-Fluciclovine and ^11^C-Methionine, consistent with similar findings for ^11^C-Methionine seen in other studies (Supplement S-3). No consistent trends were seen between uptake and either weight or height (Supplement S-1 and S-2).

### Comparison of ^18^F-Fluciclovine, ^11^C-methionine and MRI determined tumor volumes

**Fig. 4:**
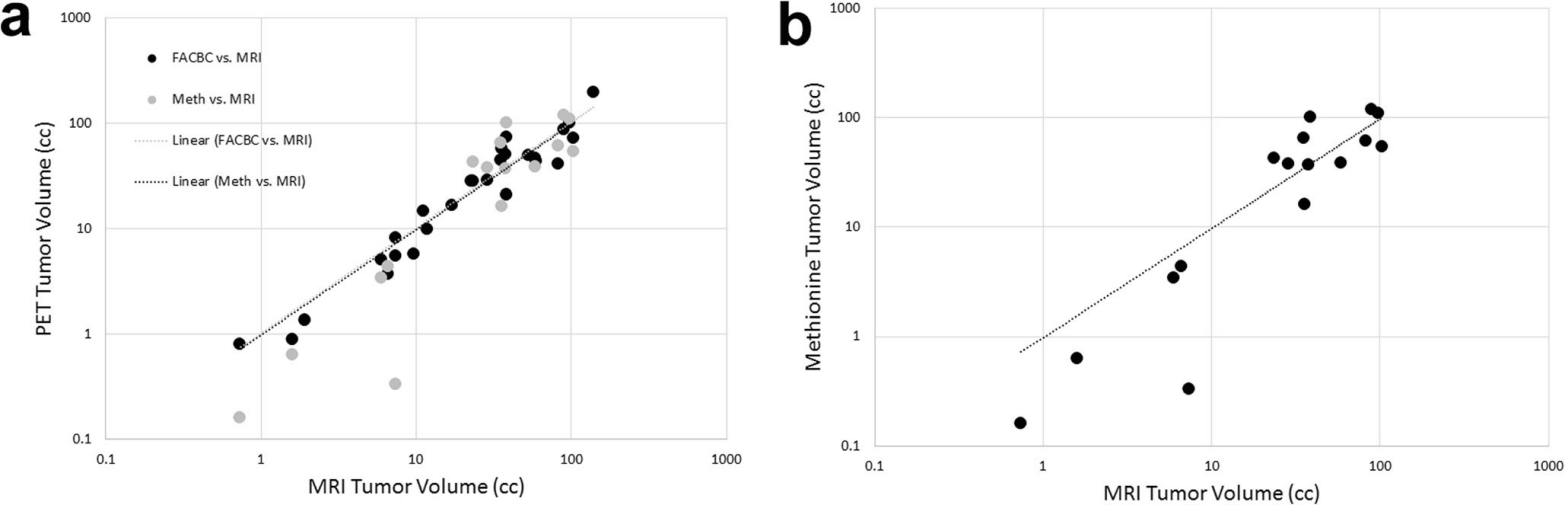
Comparison of tumor volumes as assessed on each of three image sets. **a.** Correlation between volumes determined based on T1 contrast enhanced MRI and ^18^F-Fluciclovine PET (*black circles*) and between T1c MRI and ^11^C-Methionine PET (*gray circles*). Fitted lines are forced through the origin. **b.** Comparison between the two PET derived volumes (^18^F-Fluciclovine vs. ^11^C-Methionine). Again, fitted line is forced through the origin

**Fig. 5:**
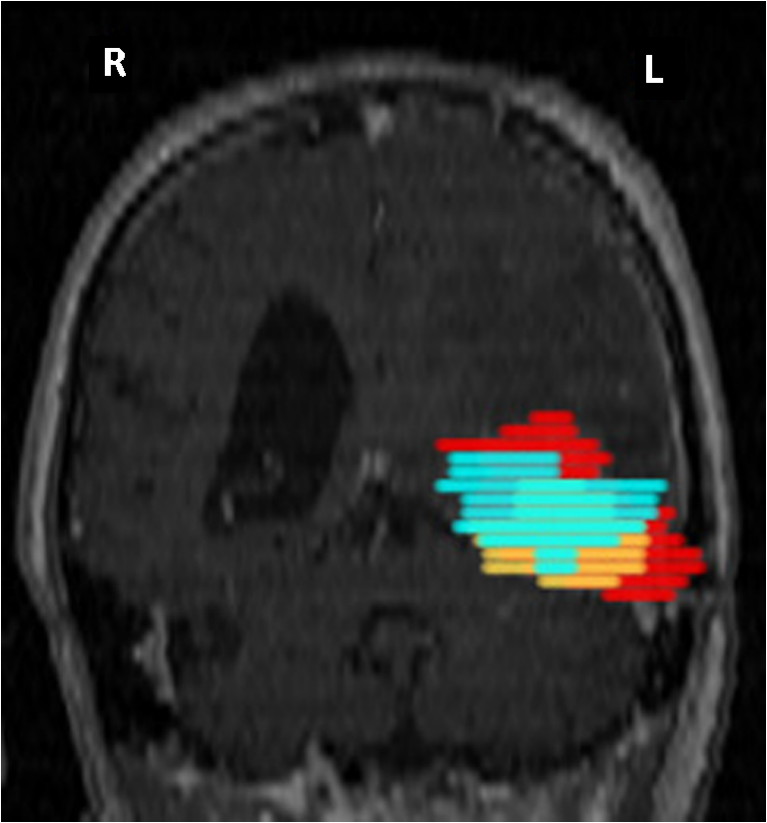
Patient 2 imaging: volumes overlap on MRI reconstruction. *Red*: MRI tumor volume, *orange*: ^18^F-FACBC tumor volume and *turquoise*: ^11^C-Methionine tumor volume. Patient 2. Male 62 years old. Primary tumor: astrocytoma grade II treated by surgery. 8 months after first recurrence treated by RT. 9 months after the first recurrence: 2nd recurrence treated by surgery and chemotherapy (Temozolomide). 3 months after the 2nd surgery: blurry vision. MRI: evocative of tumor progression. ^18^F-FACBC PET: high tumor uptake SUVmax 5.02, ratio tumor SUVmax/ Contralateral SUVmean 7.6 and ratio tumor SUVmax/ Cerebellum SUV mean 8. ^11^C-Methionine PET: high tumor uptake SUVmax 6.62, ratio tumor SUVmax/ Contralateral SUVmean 3.4 and ratio tumor SUVmax/ Cerebellum SUV mean 3.1. Progression treated by surgery. The histology report was evocative of a GBM. Adjuvant CT (CPT-11) was administrated. 6 months after, clinical deterioration and MRI progression tumor. The patient died 3 months after

Tumor volumes, as determined by ^18^F-Fluciclovine PET and contrast-enhanced MRI were highly correlated (r = 0.91) (Fig. 4a). Overlap in the volumes as measured by the Dice coefficient ranged from 36% to 87%, with a mean of 64 ± 13%. ^11^C-Methionine volumes were somewhat less well correlated to the MRI (r = 0.82) with a tendency of the ^11^C-Methionine volumes to be smaller than those based on MRI contrast for smaller lesions (<10 cc). Dice coefficients in this case varied between 7% (a lesion barely seen with ^11^C-Methionine) and 81% with a mean of 50 ± 19%. The overlap between ^18^F-Fluciclovine and MRI volumes was significantly higher than the overlap between ^11^C-Methionine and MRI volumes (*p* = 0.04). A direct comparison of the ^18^F-Fluciclovine and ^11^C-Methionine tumor volumes (Fig. 4b) was found to have a high correlation (r = 0.87)**.** The Dice values for the overlap between ^11^C-Methionine and ^18^F-Fluciclovine determined volumes ranged from 10% to 82%, with a mean of 59 ± 18% (patient’s example Fig. 5).

### Analysis of dynamic data

**Fig. 6:**
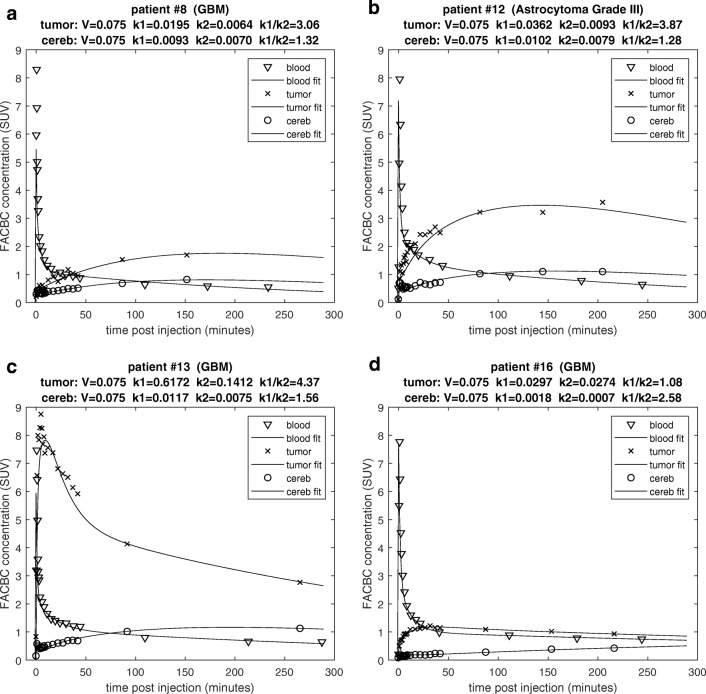
Time vs. activity profiles, along with modelled estimates. Results show ^18^F-Fluciclovine concentration in various tissues for four selected patients. Blood concentration (*triangles*), tumor (*x’s*) and cerebellum (*circles*). Parameter values associated with the model fits of the tumor and cerebellar time courses are listed in the plot titles. **a.** Patient #8. ** b**. Patient #12. **c.** Patient #13. **d.** Patient #16

Typical examples of the dynamic time-activity profiles (TACs) of ^18^F-Fluciclovine in both tumor and normal cerebellum along with the modeled estimates are shown in Fig. 6. The uptake of ^18^F-Fluciclovine into both tumor and normal brain regions was generally well described by a first-order compartmental model that included a blood space and a single reversible tissue compartment. In some instances, adding an additional exponential to the model improved the BIC value, but this generally reverted back to a single exponential if the volume of interest was adjusted so as to reduce heterogeneity within the volume. BIC values were further improved by fixing the blood volume at 7.5% for both tumors and normal brain, thereby avoiding covariance with the rate constants.

The parameter values determined by the compartmental model fits of tumor and normal cerebellar ^18^F-Fluciclovine uptake for individual patients are shown in Table 3. Not surprisingly, the tumor rate constants varied from patient to patient considerably more than did the normal brain. In general, the ^18^F-Fluciclovine tumor equilibration half-times were much shorter (57 ± 51 vs. 162 ± 139 min) and the distribution volumes considerably larger (3.2 ± 1.1 vs. 1.4 ± 0.2 ml/cc) compared to those of the cerebellum.

**Table 3:** Tumor and normal cerebellar ^18^F-Fluciclovine uptake for individual patients

Patient	Cerebellum				Tumor			
	k1 (1/min)	k2 (1/min)	Vb + k1/k2	ln(2)/k2 (min)	k1 (1/min)	k2 (1/min)	Vb + k1/k2	ln(2)/k2 (min)
2	0.0087	0.0073	1.3	95	0.1133	0.0521	2.2	13
6	0.0054	0.0042	1.4	165	0.0309	0.0100	3.2	69
7	0.0073	0.0057	1.4	121	0.1173	0.0366	3.3	19
8	0.0093	0.0070	1.4	98	0.0197	0.0065	3.1	107
12	0.0102	0.0079	1.4	88	0.0362	0.0094	3.9	74
13	0.0117	0.0075	1.6	92	0.7342	0.1674	4.5	4
14	0.0085	0.0051	1.7	136	0.0212	0.0047	4.6	148
16	0.0020	0.0014	1.5	499	0.0297	0.0274	1.2	25
mean	0.0079	0.0058	1.4	162	0.1378	0.0393	3.2	57
std	0.0030	0.0022	0.2	139	0.2443	0.0544	1.1	51
CoV	39%	38%	11%	86%	177%	139%	35%	89%
min	0.002	0.001	1.3	88	0.020	0.005	1.2	4
max	0.012	0.008	1.7	499	0.734	0.167	4.6	148

Parameter values determined by the compartmental model fits

**Fig. 7:**
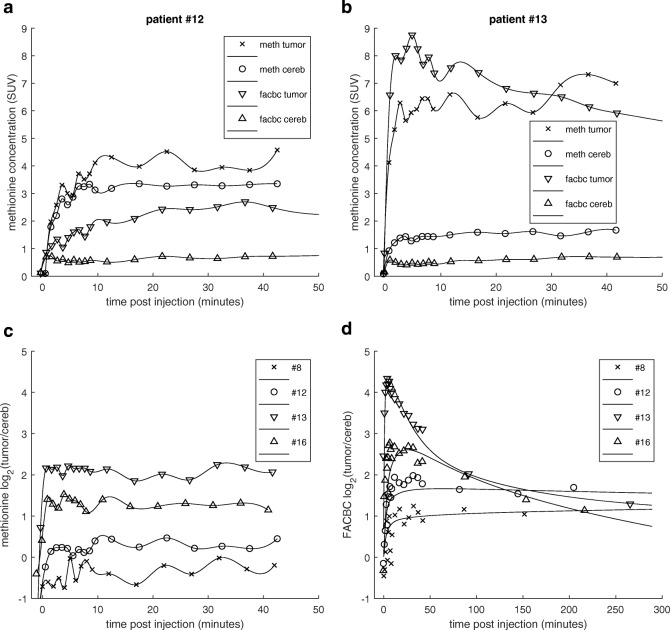
Comparison of the ^18^F- Fluciclovine and ^11^C-Methionine uptake time course over the first 45 min. Profiles are shown for two patients: #12 (**a.**) and #13 (**b.**). Tumor uptake of ^11^C-Methionine (*x’s*). Cerebellum uptake of ^11^C-Methionine (*circles*). Tumor uptake of ^18^F- Fluciclovine (*inverted triangle*). Cerebellum uptake of ^18^F-FACBC (*triangle*). Comparison of time course of tumor to normal cerebellum contrast for four selected patients: ^11^C-Methionine contrast (**c.**) and ^18^F- Fluciclovine contrast with model fitted curves (**d.**)

The analysis of the dynamic ^11^C-Methionine data was limited because blood samples were not obtained (due to patient comfort and logistical reasons related to the short half-life of ^11^C). Therefore, we did not perform any kinetic modeling and were unable to make a direct comparison of the rate constants. However, a qualitative assessment of the relative shapes of the time-activity curves can still provide some insight. The uptake of ^11^C-Methionine into normal brain was extremely fast (consistent with a rapidly equilibrating blood space) but extended to levels well above that of ^18^F-Fluciclovine, rapidly reaching a plateau. These observations suggest a much faster transport across the blood-brain-barrier (BBB) and equilibration with normal brain tissue of ^11^C-Methionine (relative to ^18^F-Fluciclovine) (Fig. 7a and 7b and Supplement S1).

Regardless of the relative tumor uptake level, ^11^C-Methionine quickly (< 5 min) reached a constant degree of contrast relative to cerebellum that extended over the majority of the 45 min imaging period (Fig. 7c). The ratio-versus-time plots for ^18^F-Fluciclovine, on the other hand (Fig. 7d), show a time dependence of image contrast with a large variation between patients.

## Discussion

^18^F-Fluciclovine (^18^F-FACBC) is an alicyclic, non-natural amino acid (anti-1-amino-3-[^18^F]fluorocyclobutane-1- carboxylic acid) containing a 4-membered ring. It was originally developed as a stable leucine analogue for brain tumor imaging [9, 18]. The mechanism of ^18^F-Fluciclovine uptake differs from that of other radiolabeled amino acids that are widely used for tumor imaging. Most amino acid transport substrates, including L-methionine, O-(2-fluoroethyl)-L-tyrosine (FET), and 3,4-dihydroxy-6-fluoro-L-phenylalanine (F-DOPA) [19] make use of the system L transporters, LAT1 (SLC7A5**)** and LAT2 (SLC7A8). In contrast, the major mediator of Fluciclovine uptake is ASCT2 (SLC1A5), with LAT1 also contributing but generally to a much lesser extent [5, 20, 21] though perhaps modulated by pH. Unlike naturally occurring amino acids, Fluciclovine is not metabolized and it is not incorporated into protein (a feature which simplifies interpretation of the images and modeling of its kinetics) [21].

Our study is one of the first evaluating ^18^F-Fluciclovine imaging in recurrent or progressive gliomas. Tumor accumulation of ^18^F-Fluciclovine was observed in all patients considered to have active disease (recurrent or progressive gliomas), even when MRI was not diagnostic. This suggests that ^18^F-Fluciclovine PET may be able to differentiate tumor recurrence/progression from post-treatment necrosis and/or inflammation (i.e. pseudo-progression on contrast MRI [6, 22]). However, we could not test this hypothesis because none of the latter were confirmed by lesion pathology, since all lesions were recurrent gliomas.

^18^F-Fluciclovine images showed high contrast, confirmed by the high tumor-to-brain ratios (tumor SUVmax-to-SUVmean of a reference normal-brain region), concordant with the first clinical studies [10–14]. Our results showed that the two ratios calculated with two different reference regions (either the contralateral normal brain tissue or the cerebellum) are highly correlated, as was previously described by Kondo et al. [10].

^18^F-Fluciclovine tumor volumes were correlated with volumes based on contrast-enhanced MRI (or CT) with extensive overlap. Compared to MRI volumes, the ^18^F-Fluciclovine PET volume was about equal for 1 patient, clearly larger for 13 patients (2 patients volumes <10% MRI volumes and 11 patients volumes >10% MRI volumes) and smaller in 13 patients (3 patients volumes < −10% MRI volumes and 11 patients volumes > −10% MRI volumes). This was a surprising finding. We were expecting that the ^18^F-Fluciclovine PET volumes would be higher than MRI volumes because in previous imaging studies in gliomas, the PET-derived amino-acid tumor volumes tended to be larger than the region of contrast enhancement [23], including in one study involving ^18^F-Fluciclovine [10]. However, in our population (recurrent/progressive disease) we did not find this to be the case. We speculate that the contrast MRI sometimes over-estimated active tumor volume in our patients, because it included treatment-induced necrosis (e.g., radionecrosis), whereas ^18^F-FACBC PET imaging primarily reflected viable progressive tumor. Unfortunately, all patients did not undergo post-imaging re-resection or biopsies of their tumors to confirm this hypothesis. The extensive BBB disruption in these patients is clearly also a potential confound (given Fluciclovine’s slow rate of transport across the intact BBB) tending to improve the correlation between the contrast MRI and Fluciclovine derived volumes.

It has been reported that most amino acid tracers (e.g., FET, MET, FACBC) do not accurately separate tumor grades based on histological subtypes [2]. Nevertheless, Parent et al. recently showed that ^18^F-Fluciclovine PET may discriminate high- and low-grade gliomas [12]. In our study we chose not to test the correlation between tumor grading and Fluciclovine uptake because the overwhelming majority of patients presented with high-grade glioma, as one might expect in a recurrent/progressive disease setting.

For 16 patients we were able to directly compare ^18^F-Fluciclovine and ^11^C-Methionine PET. The rate of ^11^C-Methionine uptake into normal brain was found to be significantly higher than that of ^18^F-Fluciclovine. This is consistent with ^11^C-Methionine’s relatively high affinity for the LAT1 transporter which is highly expressed in the endothelial cells of the brain compared to ASCT2, ^18^F-Fluciclovine’s preferred transporter. ^11^C-Methionine uptake levels achieved in the normal brain were between 1.5 and 3-fold higher than that of ^18^F-Fluciclovine. This difference in background was a major factor driving the difference in tumor contrast between ^18^F-Fluciclovine and ^11^C-Methionine, as reflected by their tumor-to-normal brain ratios. This difference was previously described by Tsuyuguchi et al. in their comparative study of six patients with initial staging of gliomas [13]. Because of this difference in contrast, ^18^F-Fluciclovine tumor volumes were easier to delineate and this too may account for ^18^F-Fluciclovine’s higher correlation and overlap with conventional imaging-derived volumes, compared to that for ^11^C-Methionine PET. Although ^11^C-Methionine is capable of being incorporated into proteins and trapped within cells, it is also conceivable that if imaging were performed at a later time post-injection, considerable washout would have occurred from the normal brain tissues to levels below that of ^18^F-Fluciclovine. Given carbon-11’s 20-min half-life, however, late imaging with ^11^C-Methionine is not practical.

An important advantage of ^11^C-Methionine PET over contrast enhanced MRI is its ability to detect infiltrating tumor even in the absence of BBB disruption. To the extent that ^18^F-Fluciclovine’s transport is hampered by an intact BBB (and conversely enhanced by a disrupted BBB), this may pose significant challenges when interpreting a Fluciclovine study. However, because the vast majority of our subjects were found to have progressive disease, we were unable to evaluate whether heightened washout from benign tissue having a disrupted BBB would have mitigated this confound.

Since LAT1 and ASCT2 are frequently upregulated in primary tumors [24] we were *not* surprised to find that the tumor SUVmax values were correlated between these two amino-acid tracers (Fig. 3a). However, it was somewhat surprising to see this correlation diminish when the tumor SUV values were normalized by either the contralateral or cerebellar normal brain mean values (Fig. 3b). The average SUV of these relatively large regions of interest should be robust, low-noise measures. Therefore, using the normal brain as a normalizer should inject a minimal amount of noise and this should more than compensate for the random biases inserted by a less than perfect body habitus normalizer. One might conclude therefore, that patient body mass (the normalizer implicit to the SUV metric we used) was near perfect. However, we also found the uptake of ^18^F-FACBC and ^11^C-Methionine to be highly correlated in the normal brain between patients (Fig. 3d). Moreover, we found that the tumor uptake of each of these tracers was correlated with that same patient’s normal brain uptake (Fig. 3c). Neither of these results is at all expected and might typically be indicative of a poor normalizer. However, if body mass were indeed a poor normalizer, one would expect there to be some residual correlation to body mass, but this was not supported by our data (Supplement S-1). Taken together, this evidence suggests that there may be some as yet unidentified parameter (e.g. endogenous amino acid blood levels) that is influencing (at least partially) the uptake of both ^18^F-Fluciclovine and ^11^C-Methionine [25].

We found that the ^18^F-Fluciclovine uptake into the normal brain when measured at 25–45 min post injection varied greatly (~8-fold with ~50% CoV’s, see Table 2), much more than we had expected. Similarly, our kinetic analysis of ^18^F-Fluciclovine uptake in these same tissues also showed equilibration half-times that varied greatly, ranging between 1.5 and 10 h with a mean of 2.7 ± 2.3 h (CoV = 86%). However, because we imaged these patients over an extended period of time (well beyond that of any previous patient study involving ^18^F-Fluciclovine), our kinetic analysis was able to find that the normal cerebellum reached a remarkably consistent distribution volume of 1.4 ± 0.2 mL/cc (CoV = 11%). This finding is potentially explained by transport rates varying as a function of variations in endogenous amino acids that are near saturation. Thus the 25–45 min time period might be too early to make a robust measurement of ^18^F-Fluciclovine uptake.

Kinetic analysis of the tumors showed the tumors to have a k1:k2 ratio (3.2 ± 1.1) that was significantly higher (*p* = 0.001) than that of normal brain (1.4 ± 0.2). Although very variable, an overall higher rate of ^18^F-Fluciclovine transport (k1) into tumors (ranging from 0.02 to 0.73 min^−1^, with a mean of 0.14 ± 0.24 min^−1^) was observed, compared to that in normal brain (0.008 ± 0.003 min^−1^). This is consistent with a higher expression of the ASCT2 transporter in gliomas compared to normal brain. It has yet to be determined which, if either, of these parameters (k1 or the k1:k2 ratio) provides more useful information. It is clear, however, that both the tumor uptake and its contrast to normal brain vary considerably as a function of the time post injection, as seen in the tumor-to-cerebellum ratio-versus- time plots (Fig. 7d). If it is determined that the k1:k2 ratio is a prognostic parameter, then it may be necessary to image later (perhaps 3 or 4 h post injection) before a simple SUV metric can be used as a robust correlate of progression or survival. Conversely, if k1 is the better parameter of interest, earlier imaging is more likely appropriate. Either way, the timing of the scan may explain the difficulty we found in demonstrating a correlation between ^18^F-FACBC uptake and survival.

Overall, ^18^F-Fluciclovine PET can detect recurrent and progressing gliomas. Compared to ^11^C-Methionine, ^18^F-Fluciclovine images have better contrast, largely due to the lower uptake of ^18^F-Fluciclovine in normal brain (lower background). The kinetic analysis of ^18^F-Fluciclovine is consistent with a linear single-compartmental model and highlights the dependence of SUV and the contrast achieved by ^18^F-Fluciclovine with time post injection. Future studies involving ^18^F-Fluciclovine should consider monitoring endogenous amino acid blood levels and should be cognizant of the changes in uptake and contrast with time post injection.

## Electronic supplementary material

ESM 1 (DOC 328 kb)
