# Supplementary material for: 18F-Fluciclovine (18F-FACBC) PET imaging of recurrent brain tumors
Source: Eur J Nucl Med Mol Imaging. 2019 Aug 15;47(6):1353–67. doi: 10.1007/s00259-019-04433-1 (PMC7188736; doi:10.1007/s00259-019-04433-1)
Supplement: Supplementary file 1 — (DOC 328 kb) [file 259_2019_4433_MOESM1_ESM.doc]

# SUPPLEMENTAL

**Figure S-1. Correlation between patient body mass and normal brain uptake of ^18^F- Fluciclovine and ^11^C-Methionine.**


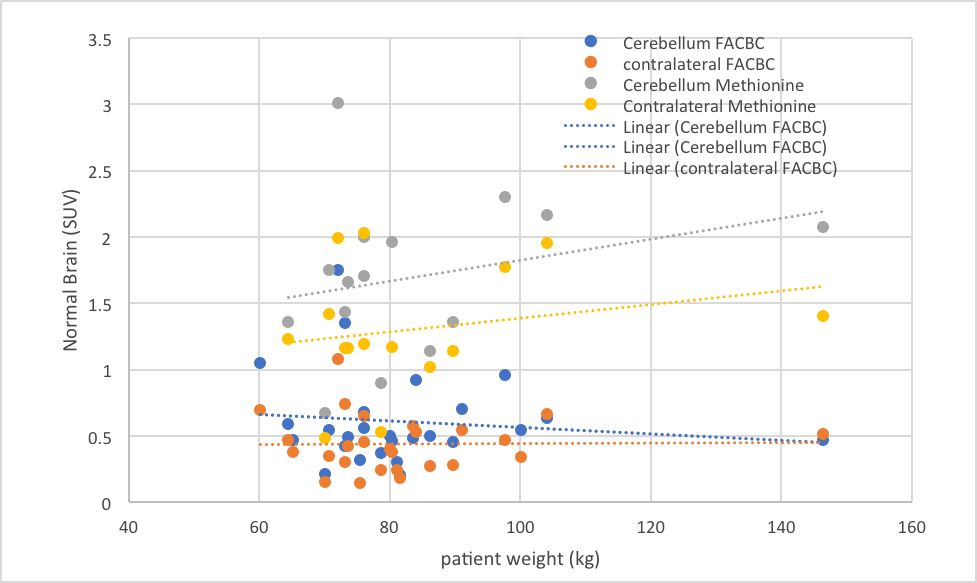


^18^F- Fluciclovine in cerebellum (blue circles) and contralateral brain (orange circles); for ^11^C-Methionine into cerebellum (gray circles) and contralateral brain (yellow circles).

**Figure S-2. Correlation between patient height and normal brain uptake of ^18^F-Fluciclovine and for ^11^C-Methionine.**


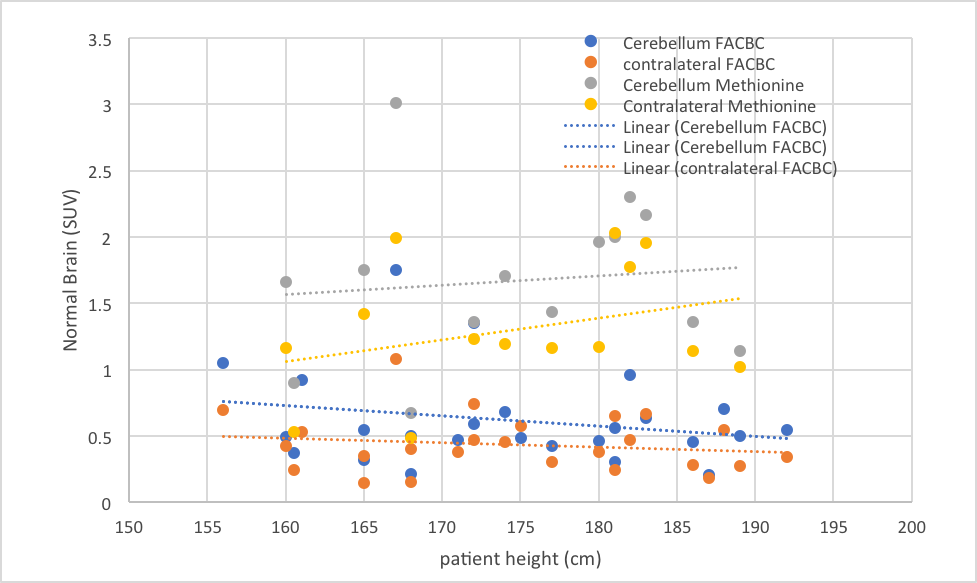


^18^F-Fluciclovine in cerebellum (blue circles) and contralateral brain (orange circles) and for ^11^C-Methionine into cerebellum (gray circles) and contralateral brain (yellow circles).

**Figure S-3. Correlation between patient age and normal brain uptake of ^18^F-Fluciclovine and for ^11^C-Methionine.**


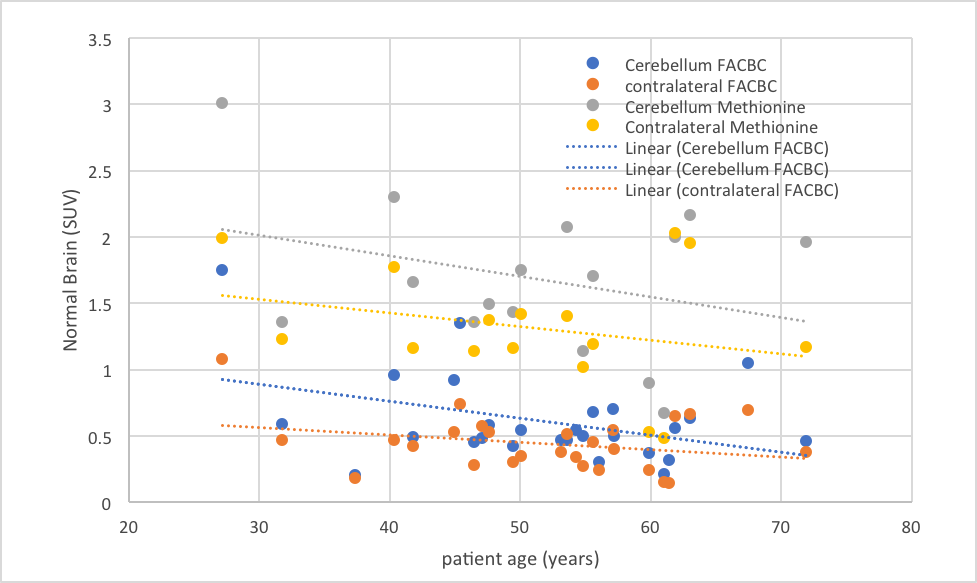


^18^F-Fluciclovine in cerebellum (blue circles) and contralateral brain (orange circles) and for ^11^C-Methionine into cerebellum (gray circles) and contralateral brain (yellow circles).

**Figure S-4. Difference in equilibration rates between ^18^F-Fluciclovine and ^11^C-Methionine in cerebellum.**

^18^F-Fluciclovine (circles) and ^11^C-Methionine (x’s) in the normal cerebellum. Example taken from a single patient (#8). Curves show concentration vs. time in tissue after removal of the blood compartment.
